# Historical Review of Stereotactic Radiosurgery in Juntendo University

**DOI:** 10.14789/jmj.JMJ22-0029-R

**Published:** 2022-10-15

**Authors:** YUTAKA NAOI

**Affiliations:** 1 Department of Radiation Oncology Juntendo University Nerima Hospital, Tokyo, Japan; 1 Department of Radiation Oncology Juntendo University Nerima Hospital, Tokyo, Japan

**Keywords:** stereotactic radiosurgery, stereotactic radiotherapy, linear accelerator, Gamma Knife

## Abstract

Juntendo University Hospital is the second hospital in Japan to start stereotactic brain irradiation using linear accelerator (LINAC) system. This report details the historical transition of stereotactic irradiation, progress of treatment technology, and change of treatment method from the beginning to the Juntendo University Hospital and Juntendo Nerima Hospital. The hospital changed the use of cobalt to the LINAC system when it was rebuilt in 1993. Total body irradiation treatment for leukemia had started around the same time. A year later, in 1994, the hospital used their LINAC systems to perform stereotactic head irradiation, otherwise known as pinpoint irradiation. In 2005, Juntendo University Nerima Hospital was opened and in September of the same year, radiation therapy using the latest model of LINAC system at that time was initiated. This was the first among all Juntendo hospitals to start intensity-modulated radiation therapy (IMRT) and image-guided radiotherapy (IGRT). In 2014, a second LINAC system for IMRT and IGRT was equipped at the Juntendo Hongo Hospital. In 2021, the LINAC systems of the Juntendo University Nerima Hospital were replaced after 15 years of usage. The new method of SRS was started using a latest LINAC systems.

In this paper, I introduce the technique and progress of SRS that I have experienced mainly in Juntendo University.

## Introduction

The Japan Radiological Society defines single stereotactic irradiation as stereotactic radiosurgery (SRS) and fractionated stereotactic irradiations as stereotactic radiotherapy (SRT), but in this manuscript, the term SRS is used without distinction. In Japan, SRS using the Gamma Knife was first started at the Tokyo University Hospital in 1991. Two years later, SRS using linear accelerator (LINAC) systems was performed at the Nagasaki University. A year later, in 1994, the Juntendo University Hospital began SRS using the LINAC systems, making it the second facility in Japan to perform LINAC-based SRS. Since then, the LINAC-based treatment method has progressed along with the technological development of the LINAC systems. I introduce the historical background and the development of SRS technique especially for Juntendo University Hospitals.

## Short history of Stereotactic Radiosurgery for brain in Japan

In 1968, the world's first SRS was started with the Gamma Knife technology at the Karolinska Hospital in Sweden (Table 1). Twenty-three years later, the first Gamma Knife surgery in Japan was started at the University of Tokyo. The successful adoption of the LINAC system for SRS by the Juntendo University, Japan, led to a rapid development of LINAC devices and subsequent increase in the number of treatment facilities in Japan.

**Table 1:** Short history of brain Stereotactic Radiosurgery in Japan

1968	Gamma Knife was bone in Karolinska Sweden
1991	The first Gamma Knife Unit was started at Tokyo University in Japan.
1993	The first Linac Radiosurgery system in Japan was started in Nagasaki Univ Hosp.
1994	The second Linac Radiosurgery was started in Juntendo Univ Hosp.
1997	Cyber knife treatment started in Japan.
1999	Mitubishi Electric co. released the C-armed Radiosurgery system.
2000	IMRT started in Japan
2004	Linac Radiosurgery covered by insurance in Japan. (63000)
2009	Linac radiosurgery with micro-multileaf collimator by Elekta synergy started at the JSDF Hosp.
2014	Gamma Knife I-con released. (Automatic Mask Syetem)
2014	Lancet oncology 2014: Stereotactic radiosurgery for patients with multiple brain metastases (JLGK0901 : Stereotactic radiosurgery without whole brain radiotherapy as the initial treatment for patients with five to ten brain metastases is non- inferior to that for patients with two to four brain metastases in terms of overall survival.
2020	Linac Radiosurgery for multiple brain metastasis started by Elekta Versa HD

In 2009, the Self-Defense Forces Central Hospital, Japan, routinely performed SRS with a micro multi-leaf and a mask system. Prior to that, SRS was performed by fixing the patient's head with an invasive metal pin. A non-invasive method of fixation was desirable to reduce the anxiety of the patients. In 2014, the Gamma Knife technology developed a mask system. The same year, a multicenter collaborative study reported positive outcomes of the Gamma Knife treatment for multiple brain metastases^1)^. Following this, the Gamma Knife treatment was actively performed in cases of multiple brain metastases to preserve the cognitive function. The history of SRS in Japan began with the Gamma Knife technology. A few years later, this was replaced by the LINAC system owing to the rapid technological development of both the software and hardware, which continues to the present date.

## The first case of SRS in Juntendo University

**Figure 1:**
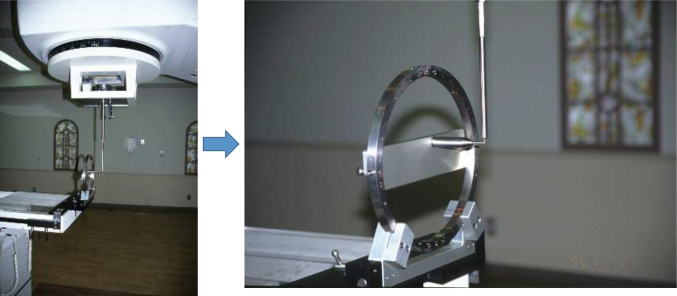
Winston-Lutz test of the first linear accelerator installed in Juntendo University

**Figure 2:**
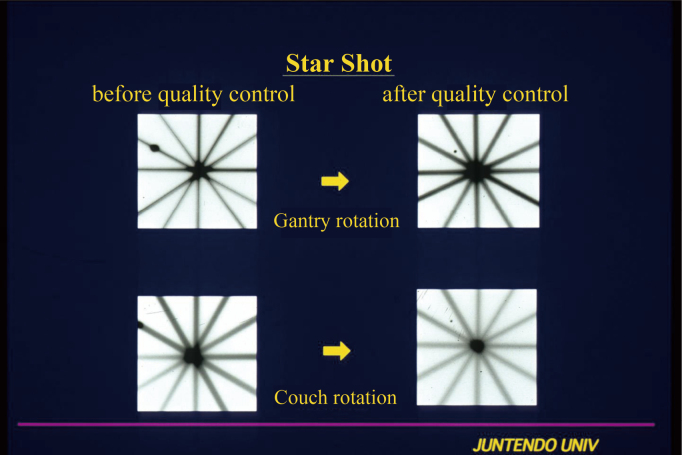
Star shot before and after quality control

**Figure 3:**
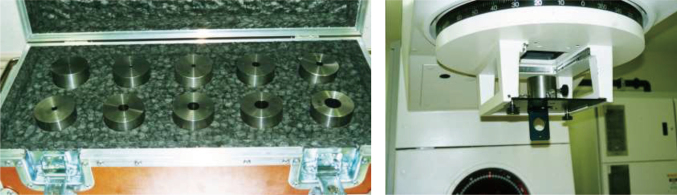
Tungsten collimator and attachment of Linac system for pencil beam

**Figure 4:**
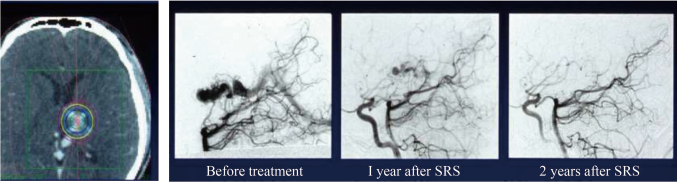
Dose distribution of cerebral AVM and post-SRS images of angiography

The first LINAC system was equipped at the Juntendo University in 1993. Figure 1 shows the Winston-Lutz test^2)^ that performs quality control of couch and gantry less than 2 mm. Figure 2 illustrates the star-shot technique. Using a 2 mm collimator, a pencil beam is emitted from the LINAC system to the low-sensitivity X-ray film. This enables accurate confirmation of the isocenters of the gantry and couch. Considerable amount of time and effort were required for the quality control step, as 30 years ago, the accuracy of the LINAC system was suboptimal. Once the quality control procedures were complete, the SRS was started. The first case of SRS in Juntendo University involved a patient with cerebral arteriovenous malformation (AVM). The nidus position was analyzed by fusing the images from enhanced computed tomography (CT) and angiography. SRS was performed with five to six directions non-coplanar stereotactic multiple arc radiotherapy. Figure 3 shows a tungsten collimator for converting X-rays to the pencil beam, which enabled the selection of an irradiation diameter from 5 mm to several centimeters according to the size of the target. After attaching the collimator to the LINAC system, the focused pencil beam X-rays irradiated the target. Two years after the SRS, the nidus had completely disappeared without complications (Figure 4).

At that time, many cases of AVM were treated by SRS in Juntendo University. 30 cases of AVM were carried out in two and a half years. These cases were reported in the journal of the Japanese Society of Radiation Oncology. Of the 14 summarized cases that followed more than 2 years, desirable results were obtained with a nidus obstruction rate of 25% in 1 year and 58% in 2 years and a nidus reduction rate of 91% in 2 years^3)^. There were no neurological side effects. Furthermore, in a report on AVM, we investigated the relationship between stagnation time of the contrast medium in angiography and nidus occlusion rate. In this study, angiographs from 25 patients with AVM were analyzed. The results showed that an AVM with a longer stagnation time of the contrast medium was more likely to be occluded^4)^. We also summarized the most common cases of brain metastasis that were treated by radiosurgery^5)^. At that time, since there was only one magnetic resonance imaging device in Juntendo University, the radiation effect of SRS was evaluated by CT images.

## SRS with non-invasive fixation of the head with a mask system

**Figure 5:**
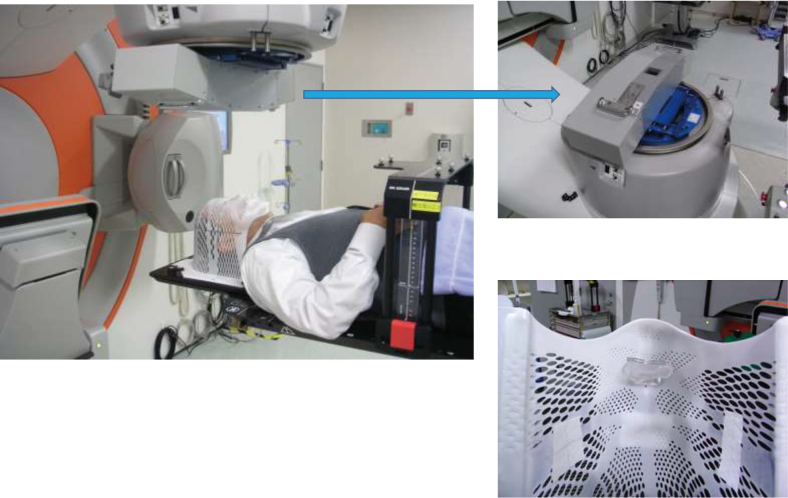
SRS with non-invasive fixation with a mask system

SRS with mask system started at 2009 while I was in the Japan Self-Defense Forces Central Hospital (Figure 5). Because of several trials, we started SRS with a non-invasive mask system instead of an invasive fixation using a head pin. The LINAC systems used the Elekta Synergy platform (Elekta AB, Stockholm, Sweden), with IGRT, 6-degree robotic couch (HexaPOD)^6)^, and 3 mm micro multi-leaf to maintain the fixation accuracy below 1 mm. HexaPOD (Elekta AB, Stockholm, Sweden) is a couch that can adjust the twisting element in addition to the XYZ position, and is a system with higher accuracy than the 3-axis couch. SRS using HexaPOD was started for the first time in Japan. Many cases have been treated with acceptable accuracy using this system^7)^.

## SRS with the latest LINAC systems

In general, 80% of SRS cases comprise brain metastases. Until around 2014, whole-brain irradiation was indicated for three to four or more brain metastases. However, in recent years, the advent of molecular targeted drugs has enabled long-term survival of patients even in advanced cancer stages. In such an era background, in recent years, cognitive decline as an adverse effect of whole-brain irradiation has become a problem.

**Figure 6:**
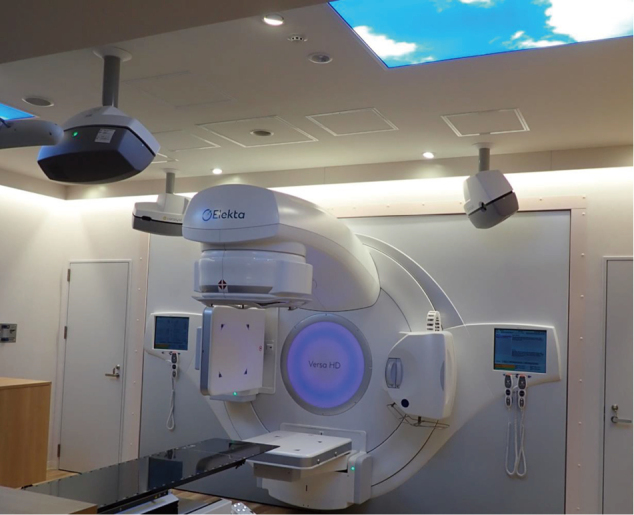
New Linac systems in Juntendo Nerima Hospital (Versa HD by Elekta corp

**Figure 7:**
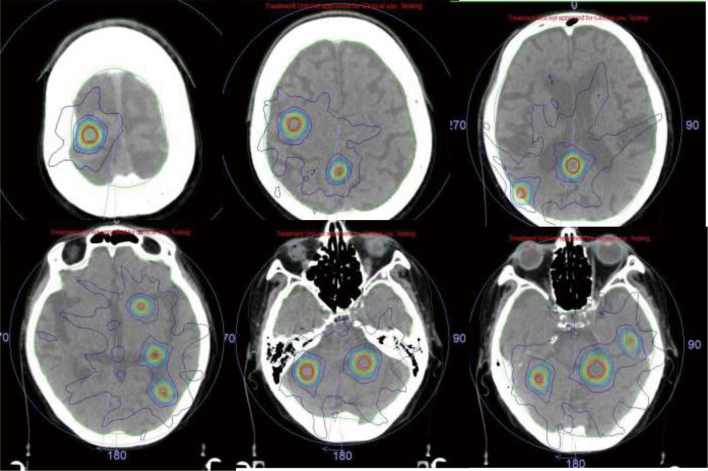
Dose distributions of SRS for 11 brain metastasis using new Linac in Juntendo Nerima Hospital

Furthermore, the results of a multicenter joint study by the Gamma Knife group^1)^ have offered a choice to perform SRS on patients with multiple brain metastases. In 2021, the LINAC systems of the Juntendo Nerima Hospital were updated to the latest model called Versa HD (Elekta AB,Stockholm, Sweden) (Figure 6). Versa HD is a highly versatile Linac capable of stereotactic radio surgery (SRS) for multiple brain tumors and high-definition volumetric modulated arc therapy (VMAT)^8)^. It is possible to obtain not only online marker-less 4D-Cone Beam CT (CBCT), but also 3D / 4D CBCT during treatment. Moreover, the treatment time is shorter than that of conventional SRS. Furthermore, the treatment accuracy has an error of 1 mm or less by using a quality control system called Catalyst (C-RAD Positioning AB, Uppsala, Sweden). Although it has become possible to perform SRS for multiple brain metastases using this systems, it is necessary to consider the indication of SRS based on the patient background. An example of SRS for multiple brain metastases is presented in Figure 7. Since this patient had a history of prophylactic whole-brain irradiation due to small-cell lung cancer, it was impossible to re-radiate the whole brain. Eleven brain metastases were effectively treated using this system by concentrating the dose distribution at each of the metastases. The average dose in the normal brain was as small as 8 Gy.

## Conclusion

SRS in Japan began with the Gamma Knife technology, but a few years later, LINAC-based SRS was started. The LINAC-based SRS was widely used, as IMRT, SRS, and conventional irradiation could be performed using the same LINAC system. In recent years, stereotactic irradiation has be selected even for multiple brain metastases if it possible. Although indications of SRS have expanded, methods of radiation therapy in future would need to be selected according to individual patient backgrounds.

## Funding

No funding was received.

## Author contributions

YN contributed to the conception, drafting the manuscript, and preparation of figures and tables.

## Conflicts of interest statement

The Author declares that there are no conflicts of interest.
